# Effect of TIMPs and their minimally engineered variants in blocking invasion and migration of brain cancer cells

**DOI:** 10.18632/oncotarget.28691

**Published:** 2025-02-28

**Authors:** Elham Taheri, Maryam Raeeszadeh-Sarmazdeh

**Affiliations:** ^1^Chemical and Materials Engineering, University of Nevada, Reno, NV 89557, USA

**Keywords:** TIMP minimal variants, glioblastoma multiforme (GBM), brain cancer, MMP inhibitors

## Abstract

Matrix metalloproteinases (MMPs) are crucial in remodeling the extracellular matrix (ECM), modulating key processes involved in cancer progression, such as migration, invasion, angiogenesis, and metastasis. The overexpression of MMPs, particularly MMP-9, is markedly observed in glioblastoma multiforme (GBM), an aggressive primary brain tumor known for its diffuse and infiltrative nature. Tissue inhibitors of metalloproteinases (TIMPs), endogenous MMP inhibitors, offer significant therapeutic potential due to their wider interaction interfaces relative to small molecule inhibitors. Here, we studied the effect of wild-type human TIMP-1 and TIMP-3 and minimal TIMP variants (mTC1 and mTC3), previously engineered for MMP inhibition, on migration and invasion of GBM cells. Our study focused on minimal TIMP variants, due to their small molecular size and potential in higher cellular uptake and delivery, to assess their potential in cell-based assays. The results demonstrated that the minimal TIMP variants, mTC1, and mTC3, effectively inhibit MMP activity underscoring their potential to limit tumor invasion and progression. Given the lethal nature of GBM and the limited efficacy of current therapies, the application of TIMPs and their engineered minimal variants represents a novel and potentially transformative approach to regulating MMP activity in GBM.

## INTRODUCTION

MMPs are a family of zinc-dependent enzymes responsible for the degradation of various ECM components, including collagen, elastin, and proteoglycans, which are crucial for cellular processes such as tissue remodeling, wound healing, and cell migration. However, excessive or unregulated MMP activity can lead to pathological conditions, such as cancer metastasis, inflammation, and tissue fibrosis [1]. MMPs have several non-ECM substrates and contribute to several aspects of cancer progression, including migration, invasion, angiogenesis, and metastasis of tumors, by degrading the ECM or interacting with growth factors and cell receptors [2–6]. MMP function is tightly regulated by other enzyme activators and inhibitors such as tissue inhibitors of metalloproteinases (TIMPs). This balance between MMPs and TIMPs is vital for maintaining ECM homeostasis, as aberrations in their interplay can contribute to diseases like tumor progression, where increased MMP activity facilitates invasion and metastasis. The coordinated action of MMPs and TIMPs is critical for both normal cellular functions and the pathogenesis of various disorders. Dysregulated enzymatic activity of specific MMPs has been implicated in promoting the progression and invasion of glioblastoma multiforme (GBM), an exceptionally aggressive primary brain tumor [7, 8]. Additionally, MMPs have been shown to play a role in the disruption of the blood-brain barrier (BBB), a process that can lead to neurodegenerative disorders [9] and contribute to other brain-related pathologies, including GBM [8, 9]. The disruption of BBB in GBM can also facilitate tumor progression and metastasis. Specific MMPs, such as MMP-9, are upregulated and associated with enhanced tumor invasion and therapeutic resistance in GBM [10–12]. Further, overexpression of MMP-3, the endogenous activator of MMP-9, was associated with higher tumor grades [13–16]. MMPs’ ability to degrade the ECM facilitates the infiltration of tumor cells into surrounding brain tissue, contributing to the diffusive and infiltrative nature of GBM [17].

Early drug discovery efforts aimed at MMP inhibitors mainly concentrated on Zn-chelating small molecules that target the catalytic site of MMPs. [18]. However, these strategies failed in late-stage clinical trials due to several challenges, including poor selectivity for MMPs over other proteases, unfavorable pharmacokinetics, and dose-limiting toxicity and side effects [19, 20]. Thus, alternative approaches other than broad-spectrum targeting of MMPs are needed. A promising new direction in therapeutic development involves engineering MMP inhibitors based on TIMPs. These MMP protein inhibitors offer enhanced selectivity and a broader interaction interface, which can be further optimized through protein engineering and design techniques. [21, 22]. TIMPs offer higher binding selectivity; thus, they have been considered potential therapeutics for targeting MMPs. Further, TIMPs could be engineered to improve binding affinity and selectivity by targeting specific MMPs through protein engineering techniques, such as directed evolution and yeast surface display [21, 23]. The four human paralogous genes encoding TIMPs, TIMP-1 to -4, have a high sequence and structure homology level, with a broad range of binding and inhibition to the MMP family. We have previously engineered minimal TIMP variants that inhibit MMPs effectively using DNA shuffling, yeast display, and directed evolution [24]. Based on previous investigations into peptides’ ability to cross the blood-brain barrier (BBB) [25, 26], these minimal TIMP variants are promising candidates for MMP inhibition in GBM cell lines with elevated MMP expression.

Understanding the complex roles of MMPs in the ECM and the BBB is essential for developing targeted therapeutic strategies to inhibit MMP-mediated invasion and improve patient outcomes in debilitating diseases such as GBM. Further, MMPs have been shown to degrade the tight junctions of endothelial cells in BBB while TIMP had a recovery role [27]. To enhance drug delivery to the brain, cell-penetrating peptides (CPPs) have been extensively studied due to their ability to penetrate biological barriers, including cell membranes and the blood-brain barrier (BBB) [28, 29]. These peptides are particularly valuable for their selective targeting capabilities through interactions with specific receptors on brain glioma tissue, and Neuropilin-1 (NRP-1), an overexpressed receptor on the surface of new blood vessels, could be a promising candidate for targeted drug delivery.

This study examined the effect of wild-type human TIMP-1 and TIMP-3 recombinant proteins and the engineered minimal TIMP variant [24] for inhibition of MMP-9 to reduce the migratory and invasive capabilities of GBM cells. MMP-9 is overexpressed in the GBM cell lines T98G and A172 [30, 31]. Our investigation was focused on two minimal TIMP variants, mTC1 and mTC3, which were engineered for inhibition of MMP-3, and MMP-9 activity—a crucial factor in the aggressive nature of GBM. Through a comprehensive evaluation of the efficacy of mTC1 and mTC3, we validated their potential as therapeutic agents and gained a deeper understanding of the MMP-TIMP role within the GBM microenvironment.

## RESULTS

MMP-9 plays a crucial role in GBM development and prognosis [32], and its inhibition has been shown to reduce the effects of GBM invasion in brain tumors [33, 34]. MMP-3, the endogenous activator of MMP-9, also plays a role in GBM invasion. We studied the effect of TIMPs (TIMP-1 and TIMP-3) and their minimal variants (mTC1 and mTC3) on cell viability, cellular uptake, migration, and invasion in two GBM cell lines, T98 and A172. These GBM cell lines have a high MMP-9 expression [30, 31, 35, 36]. The engineered minimal TIMP variants (mTC1, mTC3) are derived from screening a library of DNA shuffling within the human TIMP family (TIMP-1, -2, -3, and -4) [24]. Briefly, these peptides carry the dominant minimal MMP inhibitory regions, and they were screened toward MMP-3 and MMP-9 using fluorescent-activated cell sorting (FACS) [24].

### Minimal TIMP variant does not affect GBM cell proliferation

**Figure 1:**
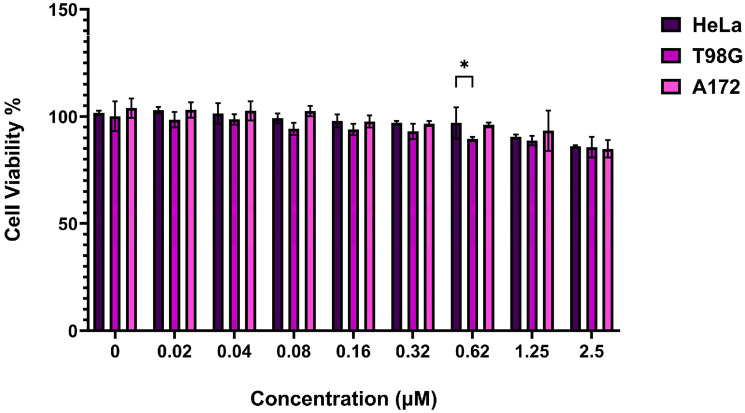
Effect of minimal TIMP variant (mTC1) on the proliferation of GBM cell lines (T98G, A172) and HeLa cell line. The experiment was performed 24 hours after adding the mTC1 peptide to the GBM cell lines. The MTT assay was performed for T98G and A172. Hela cell line was used as a control. Bar graphs represent the mean ± SEM of 4 different MTT experiments. Statistical analysis was performed using One-way ANOVA. ^*^ *P* < 0.05.

The impact of one of the minimal TIMP variants (mTC1) on cell proliferation was tested on GBM cell lines T98G and A172, as well as HeLa cells (as control) using an MTT assay. Cells were grown to confluency and treated with 0.02-2.5 µM of minimal TIMP variant, mTC1. The viability of A172 cell lines persisted unaltered more than 90% until 1.25 µM of mTC1, then decreased to 88% at 2.5 µM (Figure 1). The viability data for the T98G cell line showed that mTC1 did not affect the cell proliferation, and viability remained above 90% until 0.32 µM and then decreased to 89% and 88% at 0.62 and 1.25 µM, respectively. The 2.5 µM of mTC1 reduced the viability of the T98G cell line to 85%. The observed trend in the behavior of the HeLa cell line closely mirrored that of the GBM cell lines (Figure 1). These findings demonstrate that mTC1 has little effect on viability in GBM and HeLa cell lines at lower concentrations, with only moderate reductions at higher concentrations, suggesting a potential for targeted therapeutic use with minimal cytotoxic effects at lower doses.

### Cellular uptake

**Figure 2:**
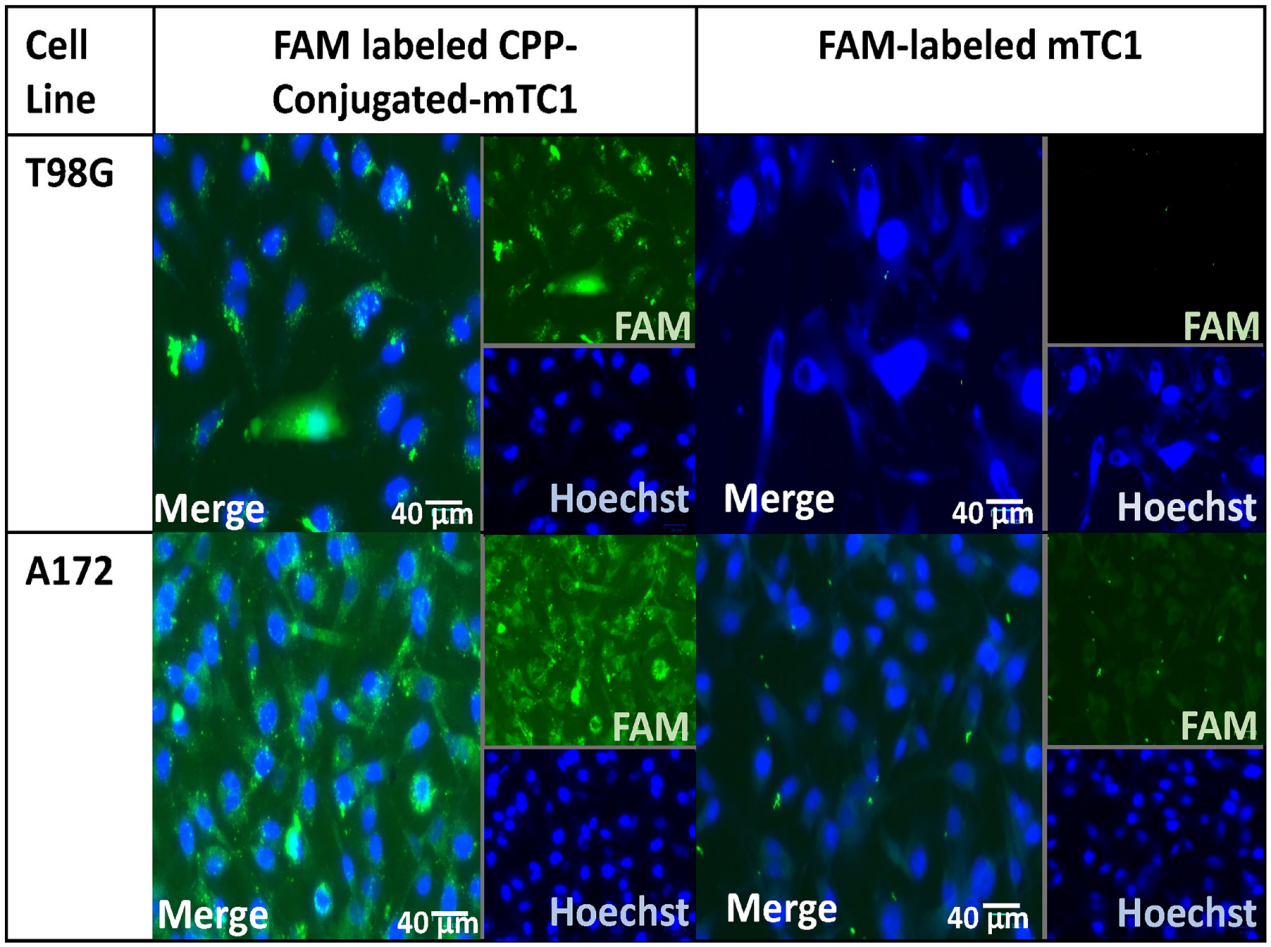
Cellular uptake of minimal TIMP variants in the T98G and A172 cell lines. Fluorescence images of T98G and A172 cell lines after incubation with FAM-labeled peptides, including mTC1, and CPP-mTC1 for 4 h. The scale bars represent 40 µm. The images are an overlay of the green and blue filters. Green, FAM-labeled peptide; blue, Hoechst-stained cell nuclei.

To improve cellular uptake of the minimal TIMPs variants, cell-penetrating peptides (CPPs) were utilized because they penetrate cells, become internalized, and breakthrough various bio-barriers, including the BBB [37–41]. One of the most promising CPPs, TAT (RKKRRQRRR), has received a great deal of research due to its high cargo delivery efficiency (antibodies, nucleic acids, and nanoparticles) and effective biological barrier penetration [42, 43]. To overcome the non-selective targeting of TAT, conjugate targeting ligands to CPPs is essential. We designed a new dual receptor recognizing cell-penetrating peptide based on the conjugation of TAT with a reverse RGD sequence (dGR), a specific ligand for integrin α_v_β_3_ family [44], to form R/KXXR/K motif at the C-terminal. The R/KXXR/K motif, which was termed the C-end Rule (CendR) phenomenon, could recognize neuropilin-1 (NRP-1) [45]. NRP-1 has a vital role in angiogenesis, and it is overexpressed in tumor cells [45, 46], especially in GBM cell lines, such as T98G and A172 [47, 48]. Therefore, the newly designed CPP (RKKRRQRRRdGR) could bind to integrin αvβ3 and NRP-1 with improved tumor targeting and tissue penetrating capabilities. The cellular uptake of the FAM-labeled CPP and mTC1-CPP peptide was evaluated on T98G and A172 cell lines using a fluorescence microscope. Fluorescence imaging demonstrated the uptake of FAM-labeled peptide in both GBM cell lines and localized in the cytoplasm, significantly higher than mTC1 in both cell lines. Nuclei were visualized using Hoechst 10 mg/ml solution in water (1:2000 in PBS) (Figure 2). This result confirmed peptide efficacy for intracellular delivery, and this validation is crucial for establishing the peptide’s potential as a viable drug candidate.

### TIMP and its minimal variants inhibit cell migration in GBM cells

**Figure 3:**
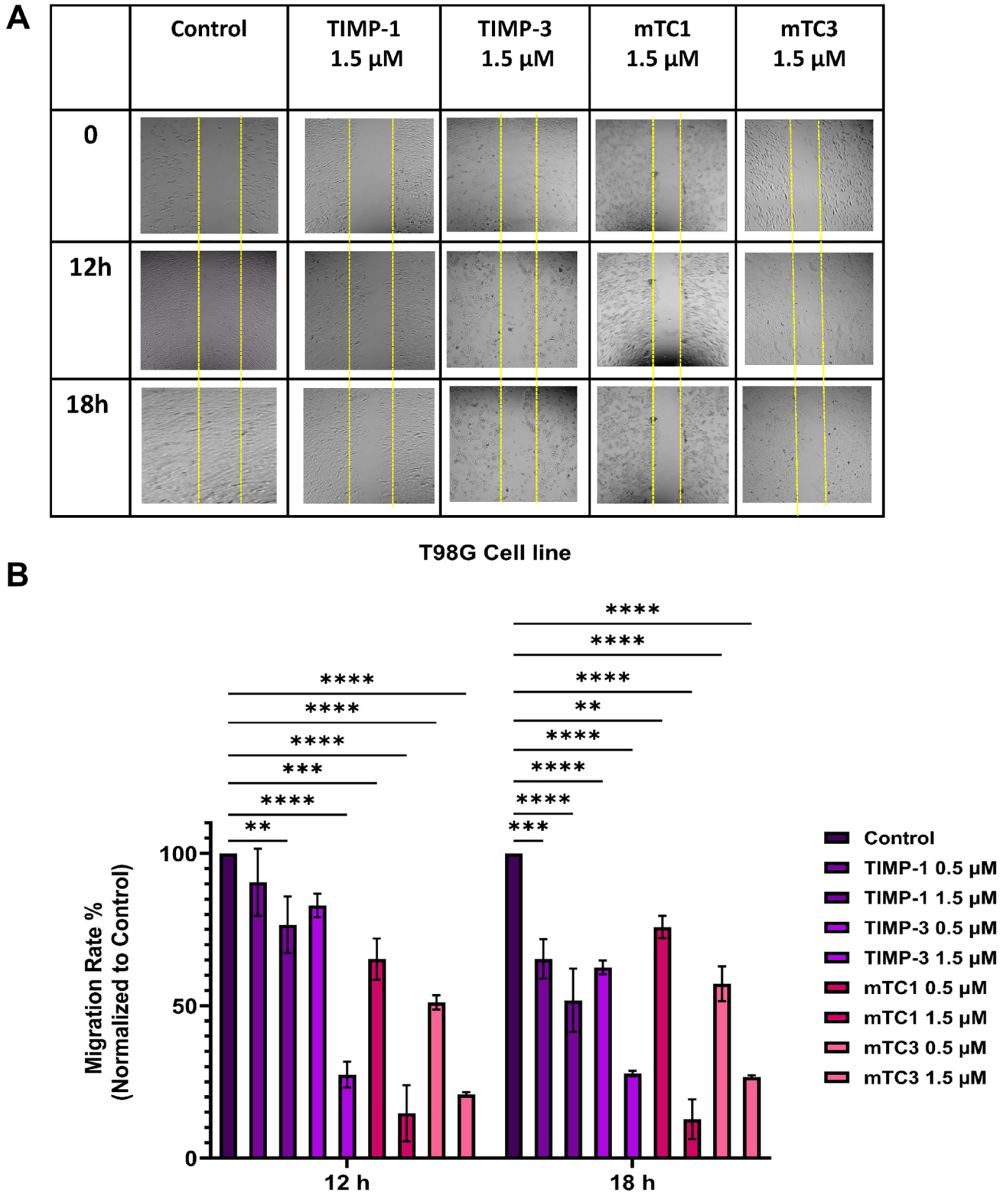
Effect of TIMPs and minimal TIMP variants on migration of the T98G cell line. (**A**) Representative images of migration T98G in the presence and absence of TIMPs and minimal TIMP variants. The cells were visualized by light microscopy using a 10X magnification lens on 0, 12 h, and 18 h, after adding 1.5 µM of TIMPs (TIMP-1, TIMP-3) and minimal TIMPs variants (mTC1, mTC3) (**B**) Calculated migration rate of T98G cell line for 0.5 and 1.5 µM of TIMPs (TIMP-1, TIMP-3) and minimal TIMPs variants (mTC1, mTC3). The created wounds were analyzed using ImageJ software and wound closure percentage was calculated (The data were normalized to control-untreated cells). The experiments were performed in duplicate; means and standard error are shown. ^****^ *p* < 0.0001, ^***^ *P* < 0.001, ^**^ *P* < 0.01, as determined by One-way ANOVA comparing inhibition in the presence of the various inhibitors versus the untreated control.

**Figure 4:**
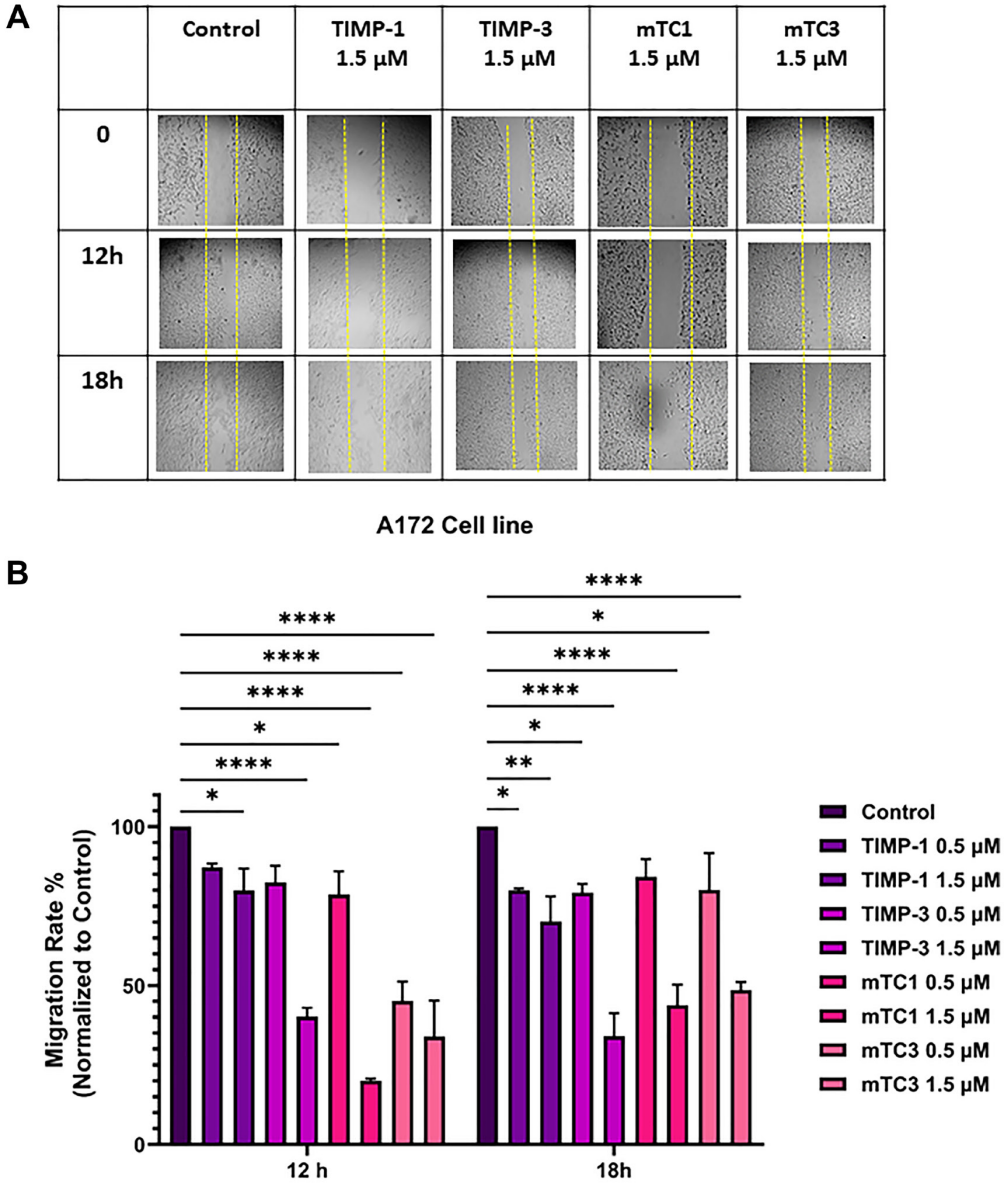
Effect of TIMPs and minimal TIMPs variants on migration of the A172 cell line. (**A**) Representative images of migration A172 in the presence and absence of TIMPs and minimal TIMP variants. The cells were visualized by light microscopy using a 10X magnification lens at 0, 12 h, and 18 h, after adding 1.5 µM of TIMPs (TIMP-1, TIMP-3) and minimal TIMPs variants (mTC1, mTC3) (**B**) Calculated migration rate of T98G cell line at concentrations of 0.5 and 1.5 µM of TIMPs (TIMP-1, TIMP-3) and minimal TIMPs variants (mTC1, mTC3). The created wounds were analyzed using ImageJ software and wound closure percentage was calculated (The data were normalized to control-untreated cells). The experiments were performed in duplicate; means and standard error are shown. ^****^ *p* < 0.0001, ^**^ *P* < 0.01, ^*^ *P* < 0.05 as determined by One-way ANOVA comparing inhibition in the presence of the various inhibitors versus the untreated control.

A wound healing assay was conducted to assess the impact of TIMPs and engineered minimal TIMP variants on GBM cell migration. As shown in Figure 3A, treatment of T98G cell with varying concentrations (0.5 µM and 1.5 µM) of mTC1 and mTC3 effectively inhibited cell migration, surpassing the inhibitory effect of TIMP-1 at the same concentrations. After a 12-hour incubation, mTC1 at 0.5 µM and 1.5 µM significantly reduced T98G cell migration to 62% and 12%, respectively, compared to the control (Figure 3B and Supplementary Figure 1). This reduction in migration was sustained after 18 hours at both concentrations. Similarly, mTC3 at 1.5 µM reduced T98G migration to 25% after 12 hours, with the effect remaining consistent at 18 hours (Figure 3B and Supplementary Figure 1). Additionally, we assessed the impact of mTC3 on migration inhibition of T98G. Our results indicate that after 12 h, mTC3 at a concentration of 1.5 µM significantly reduced T98G migration to 25% and importantly, the inhibitory effect of mTC3 on migration remained consistent after 18 h, mirroring the observations at the 12 h mark (Figure 3B and Supplementary Figure 1). To examine the impact of natural MMP inhibitors on T98G migration, we assessed two different concentrations (0.5 µM, 1.5 µM) of TIMP-1 and TIMP-3. TIMP-1 at 0.5 µM exhibited no significant effect after 12 h, while 1.5 µM of TIMP-1 reduced migration to 75%. Nevertheless, 0.5 µM and 1.5 µM of TIMP-1 significantly reduced T98G cell migration after 18 h. Our findings indicate that following an 18 h incubation period, 0.5 µM and 1.5 µM TIMP-3 significantly decreased T98G migration to 65% and 25%, respectively; however, after 12 h, 0.5 µM did not exhibit a significant effect on migration (Figure 3B and Supplementary Figure 1). The comparison between TIMP-3 and minimal TIMP variants indicated that the minimal TIMP variants, mTC1 and mTC3, significantly reduced cell migration more than TIMP-3 at a concentration of 0.5 µM in the T98G cell line, after 12 hours of treatment, however, at a higher concentration of 1.5 µM, the differences were not significant, indicating that the efficacy of the minimal TIMPs and TIMP-3 converge at higher doses (Supplementary Figure 2). The effect of TIMPs and engineered TIMP variants on A172 migration showed a similar pattern (Figure 4A). 1.5 µM mTC1 significantly reduced A172 migrated cells to 20% and 40% after 12 and 18 h, respectively. 0.5 µM mTC1 reduced migrated cells to approximately 80% after 12 h and did not show a significant effect after 18 h (Figure 4B and Supplementary Figure 3). After 12 h, both 0.5 and 1.5 µM mTC3 significantly reduced migrated cells to 45% and 35%, respectively (Figure 4B and Supplementary Figure 3). 1.5 µM TIMP-1 reduced A172 migrated cells to 75% and 65% after 12 and 18 h, respectively (Figure 4B and Supplementary Figure 3). Moreover, 1.5 µM TIMP-3 reduced A172 migrated cells to 40% and 30%, after 12 h and 18 h, respectively (Figure 4B and Supplementary Figure 3). In the A172 cell line, the difference between mTC1 and TIMP-3 at 0.5 µM was not significant, but mTC3 significantly outperformed TIMP-3 in reducing cell migration. At a concentration of 1.5 µM, mTC1 significantly reduced cell migration more than TIMP-3 (Supplementary Figure 4). These results suggest that both wild-type TIMP and minimal TIMP variants show a significant reduction in migration and invasion in GBM cell lines where MMP-9 was overexpressed. However, there was no significant improvement in the outcome between the wild-type and variants across different doses. The engineered minimal TIMP variants, particularly mTC1 and mTC3, exhibit a strong potential for inhibiting GBM cell migration similar to wild-type TIMPs, and this substantial reduction in the migration of T98G and A172 cells highlights their efficacy as potential therapeutic agents for GBM.

### Invasion tests using TIMP and engineered variants

**Figure 5:**
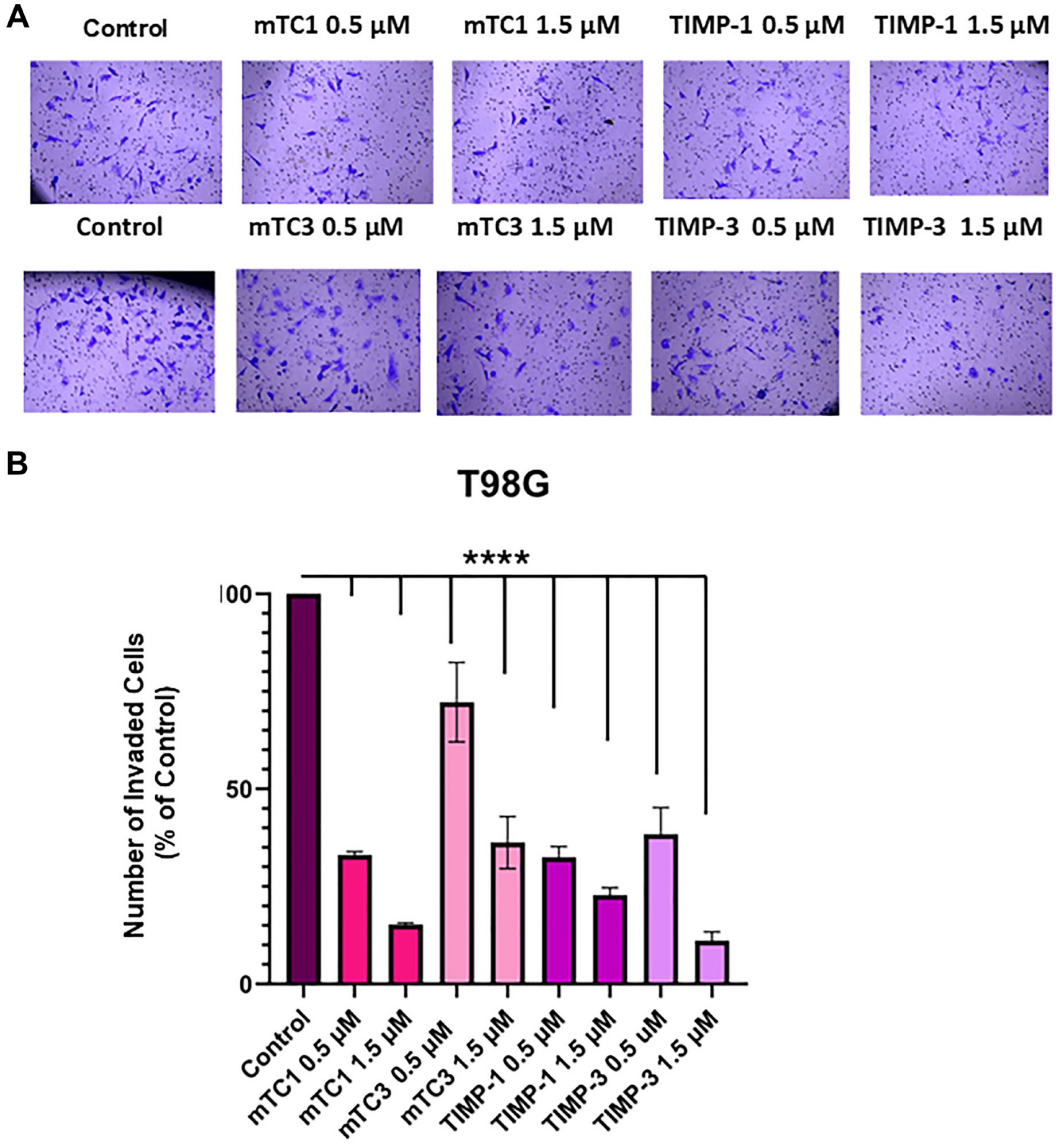
Effect of TIMPs and minimal TIMP variants on invasion of the T98G cell line. (**A**) Representative image of invasion T98G cell line in the presence and absence of TIMP-1, TIMP-3, mTC1, and mTC3. The cells were fixed in 100% methanol, stained with 2% crystal violet, and then visualized by light microscopy using an 10X magnification lens. (**B**) The percentage of invaded cells was counted using the ImageJ software and normalized to the count of untreated cells. The statistical significance was obtained using One-way ANOVA. ^****^ *p* < 0.0001.

**Figure 6:**
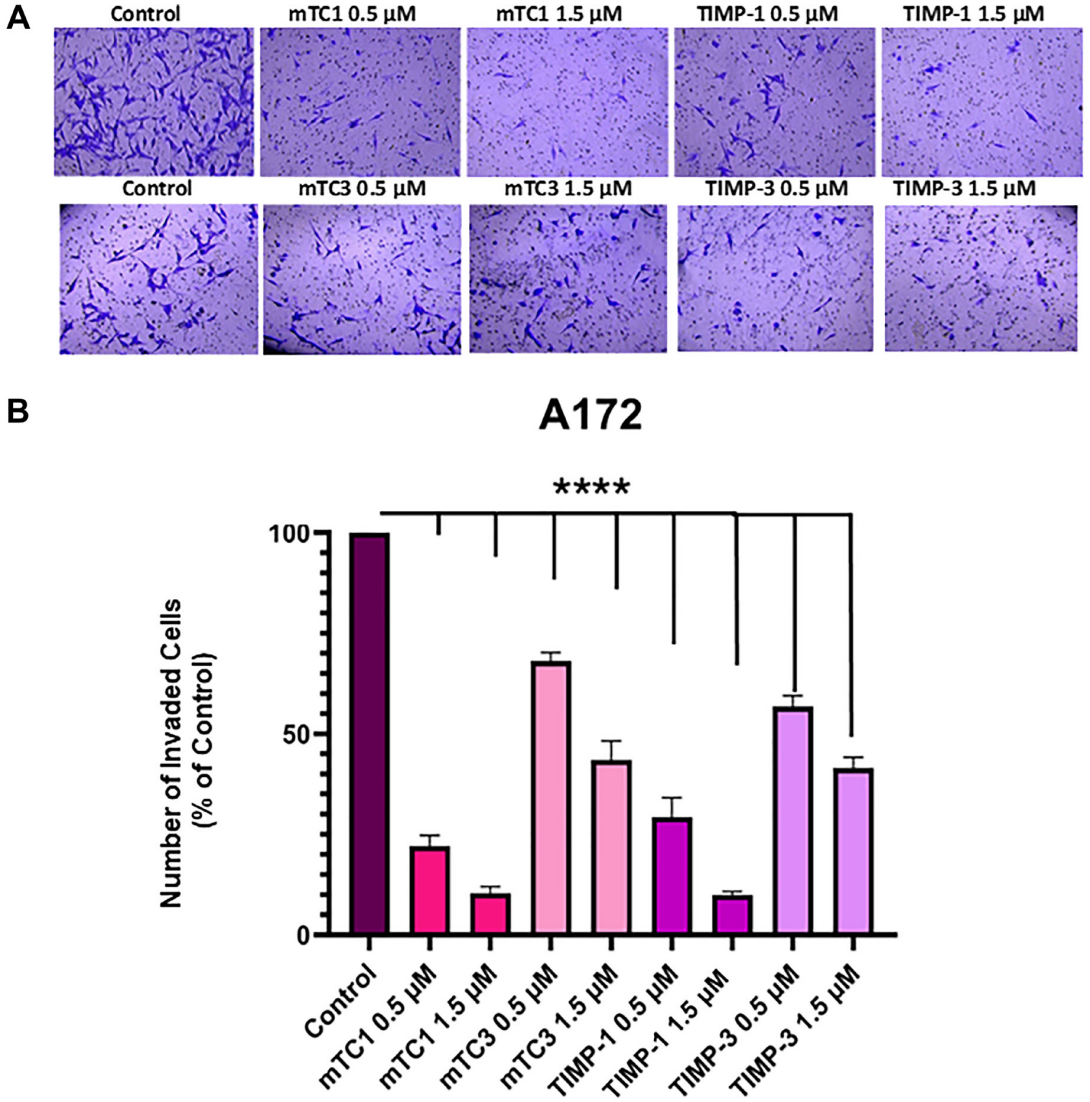
Effect of TIMPs and minimal TIMP variants on the invasion of A172 cell line. (**A**) Representative image of invasion A172 cell line in the presence and absence of TIMP-1, TIMP-3, mTC1, and mTC3. The cells were fixed in 100% methanol, stained with 2% crystal violet, and then visualized by light microscopy using an 10X magnification lens. (**B**) The percentage of invaded cells was counted using the ImageJ software and normalized to the count of untreated cells. The statistical significance was obtained using One-way ANOVA. ^****^ *p* < 0.0001.

Matrigel Transwell assays were performed to study the impact of MMP inhibition on the invasion of T98G and A172 cells. The GBM cell lines, T98G and A172 were grown on 24-well Matrigel Transwells and treated with vehicle, 0.5 µM, and 1.5 µM of TIMP-1, TIMP-3 proteins, and minimal TIMP variants. While TIMP-1 had a minimal inhibitory effect on blocking the invasion of GBM cells, the number of invasive GBM cells that traversed the Matrigel significantly decreased following mTC1 treatment compared to the control cells in a dose-dependent manner with 1.5 µM being the most effective concentration, as shown in Figure 5A. After 20 h, T98G cell invasion was decreased to 30% and 18% with mTC1 concentrations of 0.5 µM and 1.5 µM, respectively, compared to TIMP-1 with 29% and 23%, for concentrations of 0.5 µM and 1.5 µM, respectively (Figure 5B). The observed trend of invasion inhibition in the A172 cell line was consistent with that of the T98G cell line (Figure 6A), and the cell invasion was decreased to 22% and 10.3% after treatment with 0.5 µM and 1.5 µM of mTC1, respectively. (Figure 6B). Furthermore, 0.5 µM and 1.5 µM of TIMP-1 reduced invasion to 29.3 % and 9.9%, respectively (Figure 6B). These results further prove the tremendous therapeutic potential of the minimal TIMP variants.

## DISCUSSION

GBM is an aggressive brain tumor characterized by its ability to infiltrate the brain and cross the blood-brain barrier (BBB), making it particularly challenging to treat. To address these challenges, we focused on inhibiting MMPs, particularly MMP-9, which is highly expressed in GBM cell lines, as well as its endogenous activator, MMP-3. MMPs are implicated in various inflammatory diseases such as cancer, thus, finding effective therapies that target MMPs has recently become a major focus. The approach to developing inhibitors specific to a given MMP through protein engineering focuses on discovering the critical motifs in these endogenous MMP inhibitors such as TIMPs that lead to this inhibition. Techniques such as directed evolution and yeast surface display have been extensively employed to elucidate the relationship between protein sequence and function through high-throughput screening of random mutant protein libraries. The engineered minimal TIMPs generated using yeast surface display inhibited MMPs effectively. Due to the drug delivery potential of these peptides for tissue and blood-brain barrier (BBB) penetration [29], these engineered minimal TIMPs could emerge as promising candidates for MMP inhibition in GBM cell lines with elevated MMP expression, contingent upon further experimental validation.

Our results indicated that migration and invasion of GBM cell lines, T98G and A172, can be modulated by MMP inhibition, and using endogenous and engineered MMP inhibitors can reduce migration and invasion in these cell lines. Our results demonstrated that engineered minimal TIMPs, mTC1, and mTC3, effectively reduced GBM migration. Furthermore, these engineered minimal TIMPs demonstrate a similar inhibitory effect on migration to the wild-type TIMP proteins (TIMP-1 and TIMP-3), particularly notable in the T98G cell line. Transwell Matrigel assay for testing the effect of mTC1 on invasion of GBM cell lines, as a better migratory inhibitor in both cell lines, confirmed the ability of mTC1 to inhibit the invasion of both GBM cell lines. Additionally, the minimal cytotoxicity observed at lower concentrations indicates a promising therapeutic index, making these variants viable candidates for further drug development. Additionally, by integrating cell-penetrating peptides (CPPs), we achieved enhanced intracellular delivery, ensuring that these engineered variants effectively penetrate the blood-brain barrier and localize within the tumor cells.

In summary, this study provides strong evidence supporting the use of minimal TIMP variants as a novel therapeutic approach for GBM. The ability of mTC1 and mTC3 to inhibit MMP-mediated invasion and migration paves the way for future research to optimize these variants for clinical use. Future directions should include *in vivo* studies to assess the long-term efficacy and safety of these peptides, the exploration of combination therapies to enhance therapeutic outcomes, and the development of advanced delivery systems to improve targeting and penetration. Given the lethality of GBM and the limited effectiveness of current treatment modalities, advancements such as the application of minimal TIMP variants could bring novel therapeutic approaches, offering new avenues for controlling tumor spread to improve the survival and quality of life for patients battling this formidable brain tumor. By addressing the invasive and resistant nature of GBM, these innovative treatments hold the potential to significantly improve patient survival and quality of life, marking a pivotal step forward in the fight against this aggressive brain tumor.

## MATERIAL AND METHODS

### Chemical reagents

Eagle’s minimum essential medium (EMEM), Transwell with 0.8 µm pore polycarbonate membrane inserts, Matrigel basement membrane matrix, LDEV-free were obtained from Corning (Corning, Glendale, Az, USA). Dulbecco’s modified Eagle’s medium (DMEM), 0.05% trypsin/0.02% EDTA, DMSO (Dimethyl sulfoxide), MTT (3-(4,5-dimethylthiazol-2-yl)-2,5-diphenyltetrazolium bromide), Hoechst 33342, trihydrochloride trihydrate (10 mg/mL) were purchased from Life Technologies (Thermo Fisher, Waltham, MA, USA). Dulbecco’s phosphate-buffered saline (DPBS), fetal bovine serum (FBS), and 100X antibiotics (10,000 U/mL of penicillin and 10,000 μg/mL streptomycin) were obtained from Gibco (Gibco, USA). All peptides including minimal TIMP variants, CPP-conjugated minimal TIMP (mTC1), and 5-carboxyfluorescein (FAM) labeled peptides (mTC1, CPP, CPP- conjugated mTC1) were synthesized by Genscript Inc. (Piscataway, NJ, USA).

### Cell culture

The T98G glioma cell line and the HeLa cell line were generously provided as a gift by Dr. Vincent Lombardi (Pharmacology, University of Nevada, Reno) and Dr. Bahram Parvin (Biomedical Engineering, University of Nevada, Reno), respectively. A172 glioma cell line was purchased from ATCC (CRL-1620^™^, ATCC, Manassas, VA, USA). T98G and HeLa cell lines were cultured in EMEM media supplemented with 10% FBS and 1% antibiotic mixture (penicillin, 100 IU/mL and streptomycin, 100 μg/mL) in T-75 flasks (Thermo Fisher, USA). The A172 cell line was cultured in high glucose DMEM media plus 4 mM L-glutamate and 1 mM sodium pyruvate (Thermo Fisher) supplemented with 10% FBS and 1% penicillin and streptomycin mixture. The cells were incubated at 37°C in the presence of 5% CO_2_. The culture medium was changed every third day, and cells were passaged when they reached approximately 80% confluency. For sub-culturing, the cells were washed with 5 mL of DPBS, followed by the addition of 2 mL of 0.05% trypsin/0.02% EDTA, and incubated for 3 minutes until detachment occurred. Fresh culture medium was then added, and the cells were transferred to new culture flasks.

### Minimal TIMP variant amino acid sequences

The minimal TIMP peptides, mTC1 (CTCVPPHPQTAFLCTWQSLRSQIA) and mTC3 (CTCVPPHPQTAFLCTWQSLRSQIA), which derived from TIMP-1/TIMP-3 and TIMP-2/TIMP-3 sequences, respectively, were identified based on their high affinity to MMPs and then their inhibitory effect for GBM was investigated. mTC1, mTC3, mTC1-FAM, and mTC1-CPP-FAM fluorescence tag conjugated (CTCVPPHPQTAFLCTWQSLRSQIA-RKKRRQRRRdGR-FAM) peptides were synthesized by Genscript peptide services (Genscript USA, New Jersey, USA). Genscript confirmed that all peptides were obtained with >90% purity. Before use, all peptides were dissolved in the 5 µl of formic acid, then they were diluted in TNC buffer (50 mM Tris HCl, pH 7.5, 150 mM NaCl, 10 mM CaCl_2_, pH 7.5) to the final concentration of 1.5 µM for testing in the GBM cell lines.

### Cellular uptake

For the cellular uptake, 1 × 10^5^ cells of T98G and A172 cell lines were seeded on 35 mm glass base dishes (Thermo Scientific, Rochester, NY, USA) coated with 2 μg/cm^2^ fibronectin and allowed to grow for 24 h. After another 4 h incubation with 1.5 μM of fluorescence-conjugated peptide, cells were washed twice with cold PBS, fixed with 4% paraformaldehyde for 30 min at room temperature, then stained with Hoechst (10 mg/ml solution in water, diluting the Hoechst stock solution 1:2,000 in PBS) for 5 min. Finally, the cells were imaged using fluorescence microscopy (ZOE^™^ Fluorescence Imaging, Bio-Rad, Hercules, CA, USA).

### Wound healing assay

The wound healing assay was used to investigate the effects of TIMPs and minimal TIMP variants on GBM cell migration dynamics. T98G and A172 cells were grown to approximately 80% confluency, then, 1 × 10^6^ cells of each were plated in 12-well plates containing 1 mL of appropriate medium. After 24 h incubation at 37°C and 5% CO_2_, and reaching 80% confluency, straight scratch wounds were made in the middle of confluent cells in each well using a sterile P200 pipette tip along the diameter of the well. The cells were washed with Dulbecco’s phosphate-buffered saline (DPBS) (pH 7.4) to remove the debris and floating cells. After removing DPBS, cells were incubated in a medium with or without different concentrations (0.5 µM and 1.5 µM) of TIMP-1 or minimal TIMP variants for 12 and 18 h. The experiments were performed in duplicate. The initial area of the scratch or wound was taken under a light microscope immediately after creating the scratch (Inverted Microscope, Fisher Scientific, USA). This measurement provides the baseline or starting point. At subsequent time points (12, 18 hours), images of the scratch are captured again. The remaining open wound area is measured in these images using ImageJ software (National Institutes of Health, Bethesda, MD, USA). The migration distance or the percentage of wound closure was calculated using the following equation [49]:


$$\text{Wound Closure }\%=\left(\frac{{\text{A}}_{\text{t}=0}-{\text{A}}_{\text{t}}}{{\text{A}}_{\text{t}=0}}\right)\times 100$$

Where, A_t=0_ is the initial wound area immediately after creation, A_t_ is the wound area after hours of the initial scratch, both in μm^2^. A higher percentage of wound closure indicates greater migration of cells into the scratched area, reflecting enhanced cell migration and wound healing capacity. This metric provides a quantitative assessment of how effectively cells migrate and close a wound over time in response to different treatments or experimental conditions.

### Matrigel invasion assay

An *in vitro* Matrigel invasion assay was performed in a Transwell culture chamber system to assess the effect of TIMPs and minimal TIMP variants on GBM cell invasion. The filter membranes (8 μm pores, 0.33 cm^2^) were coated with 100 µL of 0.250 mg/mL Matrigel (BD Biosciences, USA) at 37°C/5% CO_2_ for 2 hours. Subsequently, 2.5 × 10^4^ GBM cells (T98G, A172) were resuspended in 500 µL of serum-free EMEM medium with or without TIMPs or minimal TIMP variants and added to the upper chamber. The lower chamber was filled with 0.75 mL of complete medium (10% FBS) as a chemo-attractant. Cells were then incubated at 37°C with 5% CO_2_ for 24 h. After incubation, non-migrated cells on the upper surface of the membrane were removed using sterilized cotton swabs. Migrated cells on the lower surface were fixed in 100% methanol and stained with 2% crystal violet. Multiple fields of cells were counted randomly in each well under a light microscope (Fisher brand^™^ Entry Level Research Grade Inverted Microscope, Fisher Scientific, USA) at 10X magnification. Data were expressed as the percentage of invasive cells compared with the control [50].

### MTT assay

The MTT (3-(4,5-dimethylthiazol-2-yl)-2,5-diphenyltetrazolium bromide) assay was performed to assess the viability of T98G/A172 and HeLa cells treated with different concentration minimal TIMP variant. Cells were seeded in 96-well plates and allowed to adhere overnight at 37°C and 5% CO_2_. The cells were then treated with various concentrations of minimal TIMP variant, media, and buffer as control solutions for 24 h while incubated at 37°C and 5% CO_2_. After treatment, 20 µL MTT solution (0.5 mg/mL) was added to each well and incubated for 4 h at 37°C/5% CO_2_. Viable cells contain NAD(P)H-dependent oxidoreductase enzymes and reduce the MTT to formazan. Formazan crystals were dissolved using 100 µL of dimethyl sulfoxide (DMSO) as a solubilization solution. The color intensity of the resulting solution is quantified by measuring absorbance at 570 nm wavelength.

### Statistical analysis

All experiments were performed at least in duplicate, and data were expressed as mean ± standard deviation (SD). Statistical analysis was performed using GraphPad Prism software. One-way analysis of variance (ANOVA) was used to determine the significance of differences between multiple groups. A *p*-value <0.05 was considered statistically significant.

## Supplementary Materials

Supplementary Figures

## Abbreviations

MMP: matrix metalloproteinase
TIMP: tissue inhibitors of metalloproteinases
GBM: glioblastoma multiforme
