# Supplementary material for: Effect of TIMPs and their minimally engineered variants in blocking invasion and migration of brain cancer cells
Source: Oncotarget. 2025 Feb 28;16:118–30. doi: 10.18632/oncotarget.28691 (PMC11870142; doi:10.18632/oncotarget.28691)
Supplement: Supplementary Figures [file oncotarget-16-28691-s001.pdf]

Effect of TIMPs and their minimally engineered variants in blocking invasion and migration of brain cancer cells

SUPPLEMENTARY MATERIALS

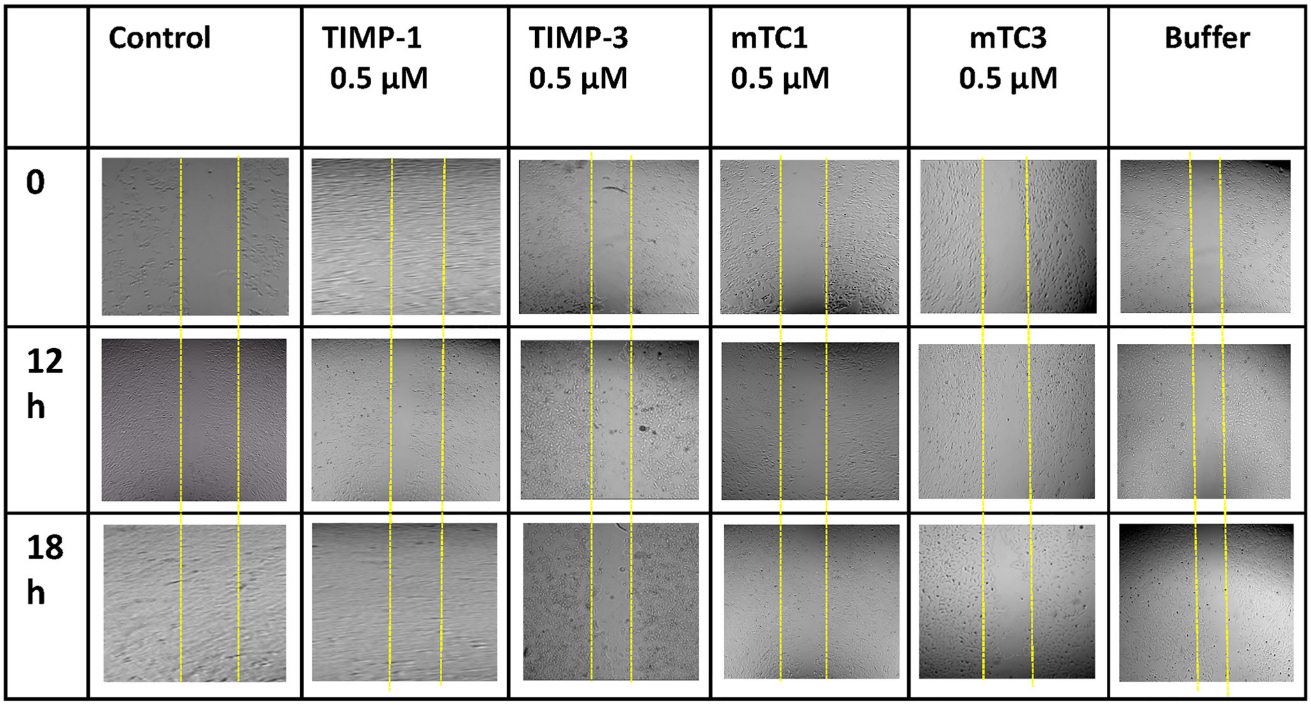

Supplementary Figure 1: Effect of TIMPs and minimal TIMPs variants on migration of the T98G cell line. Representative image for migration of T98G in the presence and absence of 0.5 µM TIMPs and minimal TIMPs variants. The cells were visualized by light microscopy using a 10x magnification lens on 0, 12 h, and 18 h, after adding 0.5 µM TIMPs (TIMP-1, TIMP-3), minimal TIMPs variants (mTC1, mTC3), buffer and media as control (Same as Figure 2A).

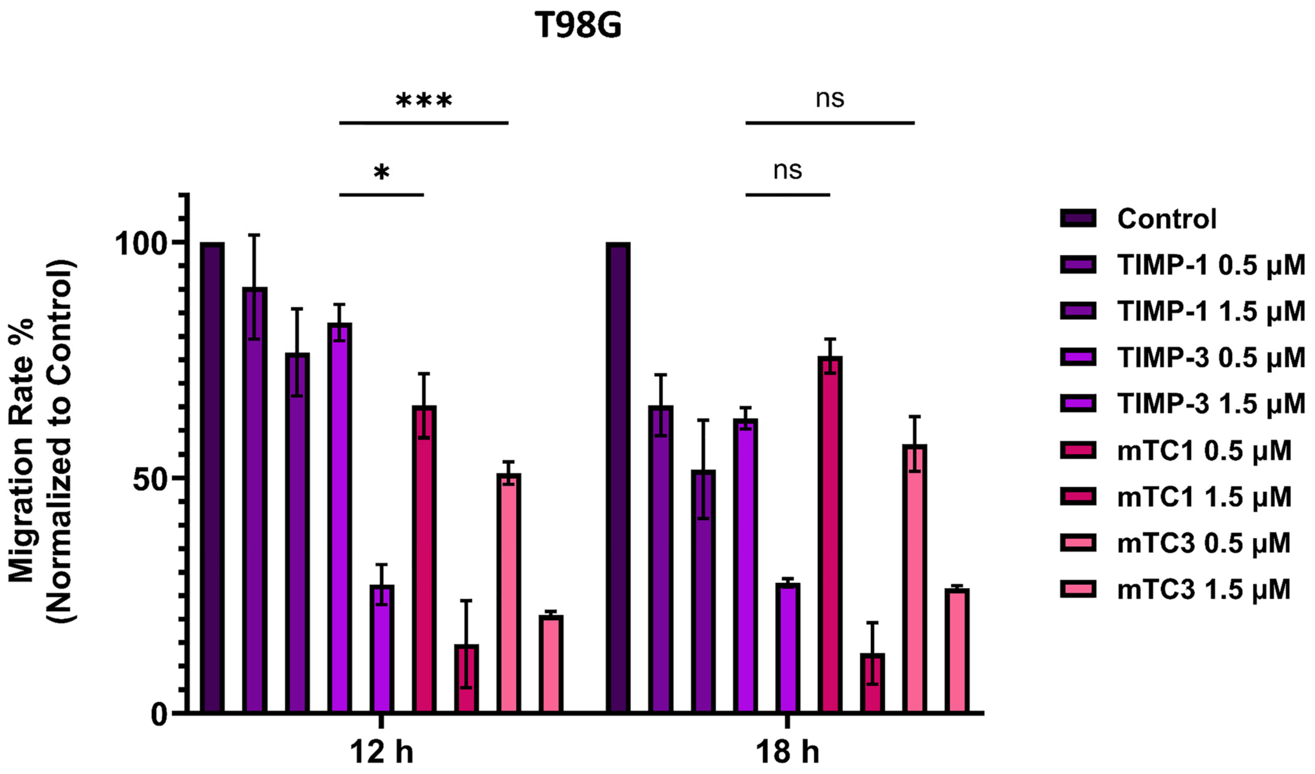

Supplementary Figure 2: Effect of TIMP-3 and minimal TIMPs (mTC1 and mTC3) on the T98G cell line. Recombinant TIMP-3 protein and engineered minimal TIMPs were compared at concentrations of 0.5 µM and 1.5 µM. The experiments were performed in duplicate; means and standard error are shown. $^{***}P < 0.001$, $^{*}P < 0.05$ as determined by One-way ANOVA comparing inhibition in the presence of the various inhibitors versus the untreated control.

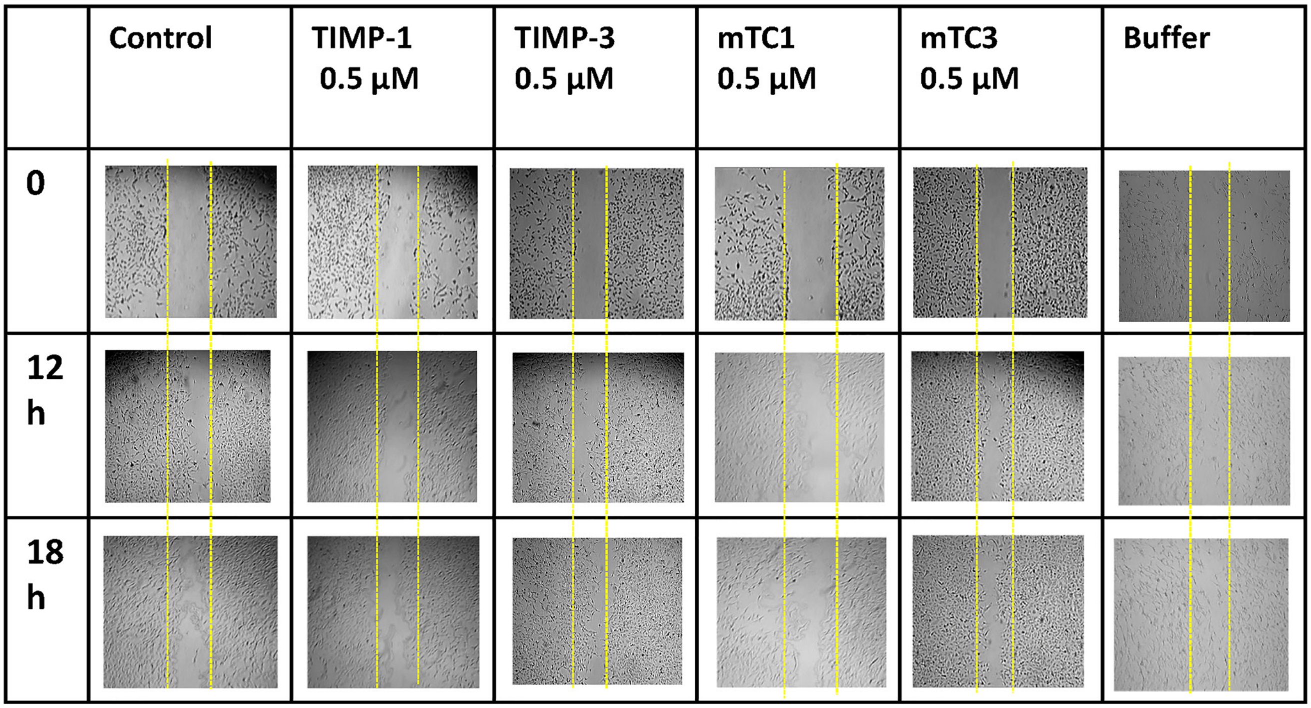

Supplementary Figure 3: Effect of TIMPs and minimal TIMPs variants on migration of the A172 cell line. Representative image for migration of A172 in the presence and absence of 0.5 µM TIMPs and minimal TIMPs variants. The cells were visualized by light microscopy using a 10x magnification lens on 0, 12 h, and 18 h, after adding 0.5 µM TIMPs (TIMP-1, TIMP-3), minimal TIMPs variants (mTC1, mTC3), buffer and media as control (Same as Figure 3A).

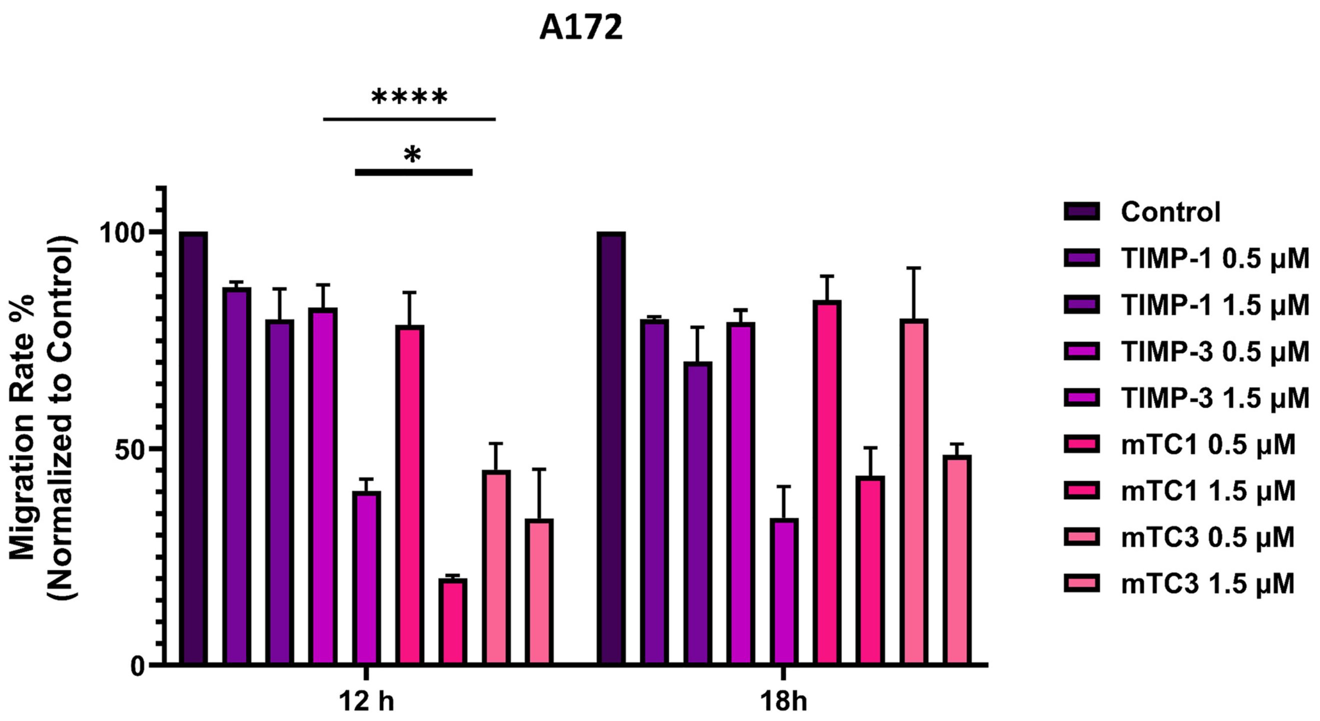

Supplementary Figure 4: Effect of TIMP-3 and minimal TIMPs (mTC1 and mTC3) on the A172 cell line. Recombinant TIMP-3 protein and engineered minimal TIMPs were compared at concentrations of 0.5 µM and 1.5 µM. The experiments were performed in duplicate; means and standard error are shown. $^{****}p < 0.0001$, $^{*}P < 0.05$ as determined by One-way ANOVA comparing inhibition in the presence of the various inhibitors versus the untreated control.
